# Bedaquiline Drug Resistance Emergence Assessment in Multidrug-Resistant Tuberculosis (MDR-TB): a 5-Year Prospective *In Vitro* Surveillance Study of Bedaquiline and Other Second-Line Drug Susceptibility Testing in MDR-TB Isolates

**DOI:** 10.1128/JCM.02919-20

**Published:** 2022-01-19

**Authors:** Koné Kaniga, Rumina Hasan, Ruwen Jou, Edita Vasiliauskienė, Charoen Chuchottaworn, Nazir Ismail, Beverly Metchock, Skaidrius Miliauskas, Nguyen Viet Nhung, Camilla Rodrigues, Soyoun Shin, Hulya Simsek, Saijai Smithtikarn, Anh Le Thi Ngoc, Jirakan Boonyasopun, Mubin Kazi, Seungmo Kim, Phalin Kamolwat, Greta Musteikiene, Catherine Ann Sacopon, Sabira Tahseen, Laima Vasiliauskaitė, Mei-Hua Wu, Shaheed Vally Omar

**Affiliations:** a Johnson & Johnson Global Public Health, Titusville, New Jersey, USA; b Department of Pathology and Laboratory Medicine, Aga Khan University, Karachi, Pakistan; c Tuberculosis Research Center, Centers for Disease Control, Ministry of Health and Welfare, Taipei, Taiwan; d Institute of Biomedical Sciences, Department of Physiology, Biochemistry, Microbiology and Laboratory Medicine, Faculty of Medicine, Vilnius University, Vilnius, Lithuania; e Department of Medical Services, Ministry of Public Health, Bangkok, Thailand; f Center for Tuberculosis, National and WHO Supranational TB Reference Laboratory, National Institute for Communicable Diseases, National Health Laboratory Services, Johannesburg, South Africa; g Reference Laboratory, Division of TB Elimination, United States Centers for Disease Control and Prevention, Atlanta, Georgia, USA; h Department of Pulmonology, Medical Academy, Lithuanian University of Health Sciences, Kaunas, Lithuania; i Vietnam Integrated Center for TB and Respirology Research, National Lung Hospital, Hanoi, Vietnam; j Department of Microbiology, P. D. Hinduja Hospital and Medical Research Centre, Mumbai, India; k The Korean Institute of Tuberculosis, Cheongju-si, Republic of Korea; l National Tuberculosis Reference Laboratory, Public Health Institution of Turkey, Ankara, Turkey; m Division of Tuberculosis, Department of Disease Control, Ministry of Public Health, Bangkok, Thailand; n Mycobacteriology Laboratory, Microbiology Unit, Central Chest Institute of Thailand, Department of Medical Services, Ministry of Public Health, Bangkok, Thailand; o National Tuberculosis Reference Laboratory, Research Institute for Tropical Medicine, Manila, Philippines; p National TB Reference laboratory, National TB Control Program, Islamabad, Pakistan; q Department of Infectious and Tropical Diseases, London School of Hygiene and Tropical Medicine, London, United Kingdom; r Yozgat Bozok University, Faculty of Medicine, Department of Medical Microbiology, Yozgat, Turkey; University of Manitoba

**Keywords:** bedaquiline, drug resistance, *Mycobacterium tuberculosis*, tuberculosis, variants, drug susceptibility testing

## Abstract

Bedaquiline Drug Resistance Emergence Assessment in Multidrug-resistant tuberculosis (MDR-TB) (DREAM) was a 5-year (2015 to 2019) phenotypic drug resistance surveillance study across 11 countries. DREAM assessed the susceptibility of 5,036 MDR-TB isolates of bedaquiline treatment-naive patients to bedaquiline and other antituberculosis drugs by the 7H9 broth microdilution (BMD) and 7H10/7H11 agar dilution (AD) MIC methods. Bedaquiline AD MIC quality control (QC) range for the H37Rv reference strain was unchanged, but the BMD MIC QC range (0.015 to 0.12 μg/ml) was adjusted compared with ranges from a multilaboratory, multicountry reproducibility study conforming to Clinical and Laboratory Standards Institute Tier-2 criteria. Epidemiological cutoff values of 0.12 μg/ml by BMD and 0.25 μg/ml by AD were consistent with previous bedaquiline breakpoints. An area of technical uncertainty or intermediate category was set at 0.25 μg/ml and 0.5 μg/ml for BMD and AD, respectively. When applied to the 5,036 MDR-TB isolates, bedaquiline-susceptible, -intermediate, and -resistant rates were 97.9%, 1.5%, and 0.6%, respectively, for BMD and 98.8%, 0.8%, and 0.4% for AD. Resistance rates were the following: 35.1% ofloxacin, 34.2% levofloxacin, 33.3% moxifloxacin, 1.5% linezolid, and 2% clofazimine. Phenotypic cross-resistance between bedaquiline and clofazimine was 0.4% in MDR-TB and 1% in pre-extensively drug-resistant (pre-XDR-TB)/XDR-TB populations. Coresistance to bedaquiline and linezolid and clofazimine and linezolid were 0.1% and 0.3%, respectively, in MDR-TB and 0.2% and 0.4%, respectively, in pre-XDR-TB/XDR-TB populations. Resistance rates to bedaquiline appear to be low in the bedaquiline-treatment-naive population. No treatment-limiting patterns for cross-resistance and coresistance have been identified with key TB drugs to date.

## INTRODUCTION

Drug-resistant tuberculosis (TB) has been declared a public health crisis by the World Health Organization (WHO) (1). In 2018, 3.4% of new TB cases and 18% of previously treated TB cases had multidrug- or rifampin-resistant TB (MDR/RR-TB). Additionally, 6.2% of MDR-TB cases were extensively drug resistant (XDR) (1). These data highlight the need for new TB drugs that are effective against drug-resistant (DR) TB and drug-susceptible (DS) TB.

Use of phenotypic drug susceptibility testing (pDST) to guide anti-TB therapies minimizes the risk of developing resistance and maximizes the effectiveness of treatments (2, 3). Two main approaches for Mycobacterium tuberculosis routine pDST and drug resistance surveillance (DRS) are the solid-based agar proportion (AP) method and the liquid-based mycobacteria growth indicator tube (MGIT) (4). These methods generate results after an average time of up to 4 weeks for subculture and up to 4 weeks for pDST (AP) and 8 to 14 days for subculture and 8 to 14 days for pDST (MGIT) from sputum-positive culture, with results interpreted based on the single critical concentration (CC) of the drug (5). Other methods increasingly used for M. tuberculosis pDST are the 7H10 or 7H11 agar dilution (AD) and 7H9 broth microdilution (BMD) methods, which, in contrast to AP and MGIT, test a range of drug concentrations. The results are reported as the MIC, defined as the lowest concentration of a drug that inhibits M. tuberculosis growth *in vitro* (3). Although not used in routine practice, AD MIC and BMD MIC can generate more granular data for determining trends of decreased susceptibility over time during a DRS and the epidemiological cutoff value (ECV) for setting the breakpoint of a drug.

Bedaquiline (BDQ), a diarylquinoline antimycobacterial drug that inhibits ATP synthase of M. tuberculosis (6), is indicated as part of combination therapy in adult and pediatric patients (≥5 years, weighing ≥15 kg) with pulmonary MDR-TB. Use of BDQ-based regimens for the treatment of MDR-TB has considerably improved treatment outcomes (7–12). However, a number of resistance-associated variants (RAVs) may decrease susceptibility to BDQ (13–20), including *Rv0678* RAVs, which lead to low-level BDQ resistance and cross-resistance with clofazimine (CFZ) (14, 15, 18, 20–23), and *atpE* RAVs in the BDQ target (19, 24).

This paper reports the results from the Bedaquiline Drug Resistance Emergence Assessment in MDR-TB (DREAM) program to assess the susceptibility of BDQ treatment-naive patients’ MDR-TB isolates to BDQ over a 5-year period in 11 countries by the BMD and AD MIC methods. The study determined whether (i) any revisions were required to the MIC quality control (QC) ranges for BDQ and other anti-TB drugs from a multilaboratory, multicountry, reproducibility study conforming to Clinical and Laboratory Standards Institute (CLSI) Tier-2 criteria (25, 26; also see the supplemental material); (ii) ECVs for BDQ were in agreement with those in the external quality assessment (EQA) study (27); and (iii) there was cross-resistance between BDQ and CFZ, coresistance to BDQ and linezolid (LZD), or coresistance to CFZ and LZD.

## MATERIALS AND METHODS

### Study design.

This was a prospective *in vitro* study conducted over a 5-year period (2015–2019) in India, Lithuania, Pakistan, the Philippines, South Africa, South Korea, Taiwan, Thailand, Turkey, Vietnam, and the United States (Centers for Disease Control and Prevention).

### Study materials.

For AD MIC testing, laboratories were provided with BDQ active pharmaceutical ingredient lot number A13HB2843 (Janssen, Beerse, Belgium). For BMD MIC testing, custom-made frozen polystyrene microtiter plates containing BDQ and other anti-TB drugs and ancillaries were supplied by Thermo Fisher Scientific (Oakwood Village, OH). Each lot of frozen microtiter plates was tested by an independent laboratory prior to use in the study to ensure the performance of the plates was according to previously published QC parameters (25, 26). All other reagents, including Middlebrook 7H10 and 7H11 agar, oleic acid albumin dextrose catalase, M. tuberculosis H37Rv strain (American Type Culture Collection number 27294), and standard medium (e.g., Lowenstein-Jensen) routinely used to grow M. tuberculosis in the laboratory were sourced at the country level.

### Microbiology methods.

M. tuberculosis isolates were collected from BDQ treatment-naive patients between 1 January 2015 and 31 July 2019, but only MDR-TB isolates from the 11 countries were included in BDQ DREAM program analyses. Isolates were included in the analyses based on the criteria defined in Table S1 in the supplemental material. The distribution of isolates per country is shown in Fig. S1. MIC determination of BDQ by AD and BMD was performed according to methods previously described for Tier-2 studies (25). An M. tuberculosis QC strain (H37Rv) was included in each testing and was required to be within previously published QC ranges for BDQ testing (25).

### WGS.

A total of 78 available isolates confirmed to be resistant to BDQ either by AD or BMD were analyzed at the WHO Supranational Reference Lab, National Institute for Communicable Diseases in Johannesburg, South Africa. DNA was isolated and submitted for whole-genome sequencing (WGS). DNA extraction was performed using either the automated bead-based Nuclisens EasyMag platform or the cetyl trimethylammonium bromide (CTAB) method. WGS and bioinformatic analysis was performed as previously described (28; also see the supplemental material). The genetic targets investigated included the intergenic regions between *mmpL5* and *Rv0678*, *Rv0678*, *atpE*, *mmpL5*, *mmplS5*, *pepQ*, and *Rv1979c*. Lineage was further assigned to these isolates using WGS as described previously (29).

### Data management.

Each laboratory was defined by its country of origin. Consistent capture and reporting of data between all laboratories was overseen by the principal investigators, who were provided with standardized data collection forms. Inconsistent data (such as MIC not within the specified dilution range, comma as decimal separator, and erroneous dilution) were queried, and the investigator was required to resubmit and update the file for the final analyses. The sponsor’s clinical microbiologist assessed the quality of the data set from each laboratory and performed a final QC check.

### Statistical and microbiological analyses.

For the range of dilutions used in the study, the frequency and cumulative frequency of MIC distribution were calculated using SAS software version 9.2 (SAS Institute). For MIC values preceded by a less-than sign, the lower-end MIC value of the range was reported with a less-than-or-equal-to sign. For MIC values preceded by a greater-than sign, the MIC value was reported as greater than or equal to the next dilution (e.g., >1 was reported as ≥2). MIC distribution histograms were produced from the MIC frequency tables, and ECVs were derived by visual inspection of the histograms as the drug concentration that delineated the wild-type from the non-wild-type population. When there is no clear separation of the wild-type from the non-wild-type population, the visual inspection of the histogram becomes subjective. In that case, an iterative nonlinear regression on expanding subsets, known as ECOFFinder, was also used to estimate ECVs (30), with a 97.5% cutoff from ECOFFinder used as the ECV.

For the AD MIC methods, the combined data for 7H10 and 7H11 agars are reported, since there are no meaningful differences between the results obtained using these two media (25). For BDQ, ECVs were used to confirm the breakpoints validated in the sponsor’s EQA study (27). For other drugs tested in this study, ECVs were used as surrogate interpretative criteria to determine the susceptibility rates of MDR-TB isolates. Categorical analyses between breakpoints derived from the 7H9 BMD MIC and AD MIC methods were performed. The evolution of BDQ MIC over the last 5 years (2015 to 2019) was also assessed.

Analyses were performed on the data set stratified by MDR-TB, MDR_H&R_-TB (MDR-TB limited to isoniazid and rifampin resistance), pre-extensively drug-resistant (pre-XDR)_FQ_-TB (MDR-TB with resistance to any fluoroquinolone tested), pre-XDR_SI_-TB (MDR-TB with resistance to any second-line injectable tested), and XDR-TB (MDR-TB with resistance to any fluoroquinolone and any second-line injectable tested) (Table S1). The resazurin microtiter assay critical concentrations for first- and second-line drugs were used to define the M. tuberculosis resistance subtypes (31–35). Capreomycin (CAP) and kanamycin (KAN) critical concentrations (2.5 μg/ml) were extrapolated to 4 μg/ml (the next dilution in the CLSI dilution scheme) (36). A small set of DS-TB found in the data set was also analyzed for susceptibility to BDQ only.

## RESULTS

### Tier-3 QC ranges.

**FIG 1:**
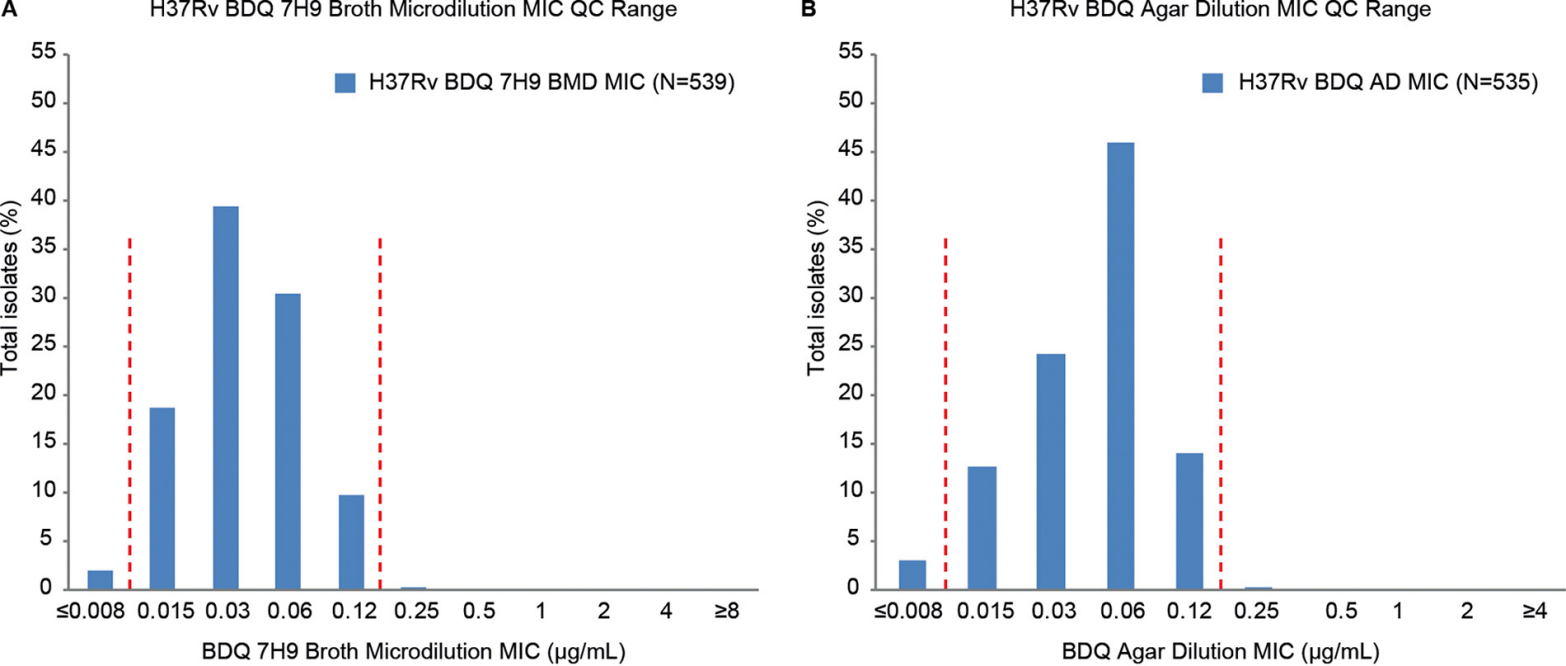
Bedaquiline MIC quality control ranges for H37Rv by the 7H9 broth microdilution (A) and agar (B) dilution methods. Dashed lines indicate the BDQ QC ranges established through Tier-3 studies. BDQ QC ranges determined in the Tier-2 studies were 0.015 to 0.06 for the 7H9 BMD MIC and 0.015 to 0.12 for the AD MIC (25).

Each day of testing clinical isolates, the M. tuberculosis QC strain H37Rv was also tested to accumulate more real-world QC data. The BDQ AD MIC QC range for the H37Rv strain was 96.8% (518/535) within the established Tier-2 QC range compared with 88.3% (476/539) for BMD MICs. Therefore, the QC range for BMD MICs was adjusted to include at least 95% of repeats while remaining within a 4-dilution range (25). The result of the Tier-3 QC of BDQ for the BMD MICs is 0.015 to 0.12 μg/ml (between the vertical lines in Fig. 1A), which includes 98.0% (528/539) of the data.

### Confirmatory phenotypic DST and whole-genome sequencing of putative BDQ-R isolates.

All nonduplicate clinical isolates with either BDQ BMD MIC of >0.12 μg/ml or AD MIC of >0.25 μg/ml were retested by BMD and MGIT at a CC of 1 μg/ml, and WGS was performed. Due to the COVID-19 pandemic, only 78 viable isolates were available for retest by BMD, MGIT, and WGS analysis. No mutations were found in the BDQ target *atpE* and *pepQ* genes; 10/78 (12.8%) isolates with BDQ BMD MIC of ≤0.12 μg/ml were BDQ susceptible (BDQ-S) by MGIT, and 10/10 (100%) were wild type for the *Rv0678* gene. This finding supports BDQ BMD MIC of ≤0.12 μg/ml as the BDQ-S breakpoint; 23/78 (29.5%) isolates with BDQ BMD MIC of ≥0.5 μg/ml were BDQ-resistant (BDQ-R) by MGIT, and 21/23 (91.3%) had nonsynonymous nonsilent mutations in the *Rv0678* gene. This finding supports a BDQ BMD MIC of ≥0.5 μg/ml as the BDQ-R breakpoint. There were 45/78 (57.7%) isolates with BDQ BMD MIC of 0.25 μg/ml, of which 17 (37.8%) were BDQ-R by MGIT; 10/17 58.8%) had *Rv0678* RAVs and 7/17 (41.2%) were wild type for *Rv0678*. The remaining 28/45 (62.2%) were BDQ-S by MGIT; 2/28 (7.1%) had *Rv0678* RAVs, and 26/28 (92.9%) were wild type for *Rv0678*. These data show that a BDQ BMD MIC of 0.25 μg/ml cannot consistently define M. tuberculosis clinical isolates as either phenotypically BDQ-S or BDQ-R by MGIT. Similarly, a BDQ BMD MIC of 0.25 μg/ml cannot consistently define M. tuberculosis clinical isolates as either genotypically BDQ-S or BDQ-R based on *Rv0678* as a marker. Thus, a BDQ BMD MIC of 0.25 μg/ml fits the typical definition of the European Committee on Antimicrobial Susceptibility Testing (EUCAST) area of technical uncertainty (ATU) or CLSI intermediate (I) category, although these proposed breakpoint categories are yet to be validated or approved for mycobacteria. Similar trends were seen for BDQ AD; however, the correlation between BDQ AD MIC and MGIT was weaker.

### BDQ BMD MIC distribution and ECV for MDR-TB isolates.

**FIG 2:**
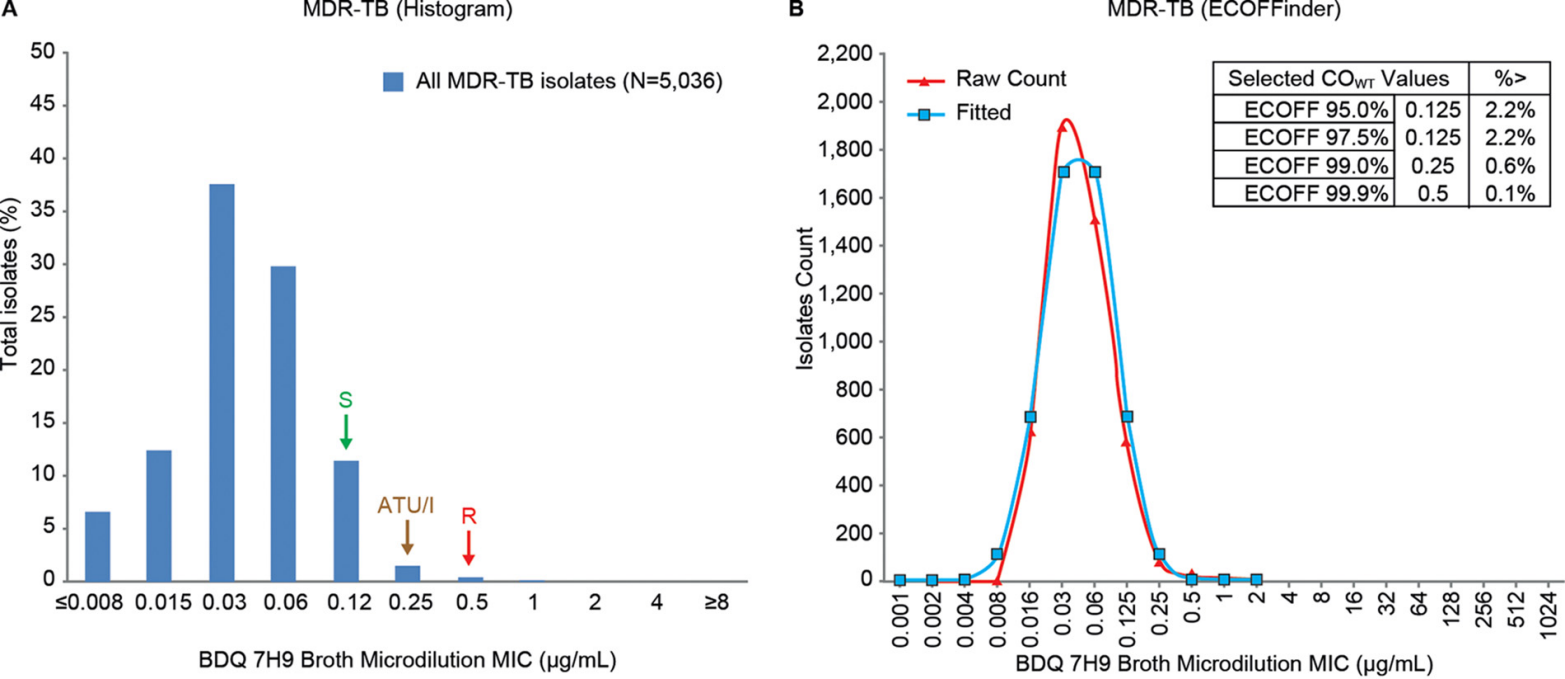
Bedaquiline 7H9 broth microdilution MIC distribution for MDR-TB clinical isolates. Susceptible (S), area of technical uncertainty (ATU)/intermediate (I), and resistant (R) breakpoints are indicated by arrows.

The BDQ BMD MIC distribution for all MDR-TB isolates was unimodal, with a trailing tail and a peak of 0.03 μg/ml (Fig. 2A), which was similar to the H37Rv MIC distribution (Fig. 1A). The BDQ BMD MIC distribution delineated an ECV of 0.12 μg/ml (Fig. 2A, indicated by S), which was consistent with the 97.5% ECV (0.125 μg/ml) determined from ECOFFinder (Fig. 2B) and previous findings (27). Based on this analysis, 2.2% of isolates had MICs above the ECV.

### BDQ AD MIC distribution and ECV for MDR-TB isolates.

**FIG 3:**
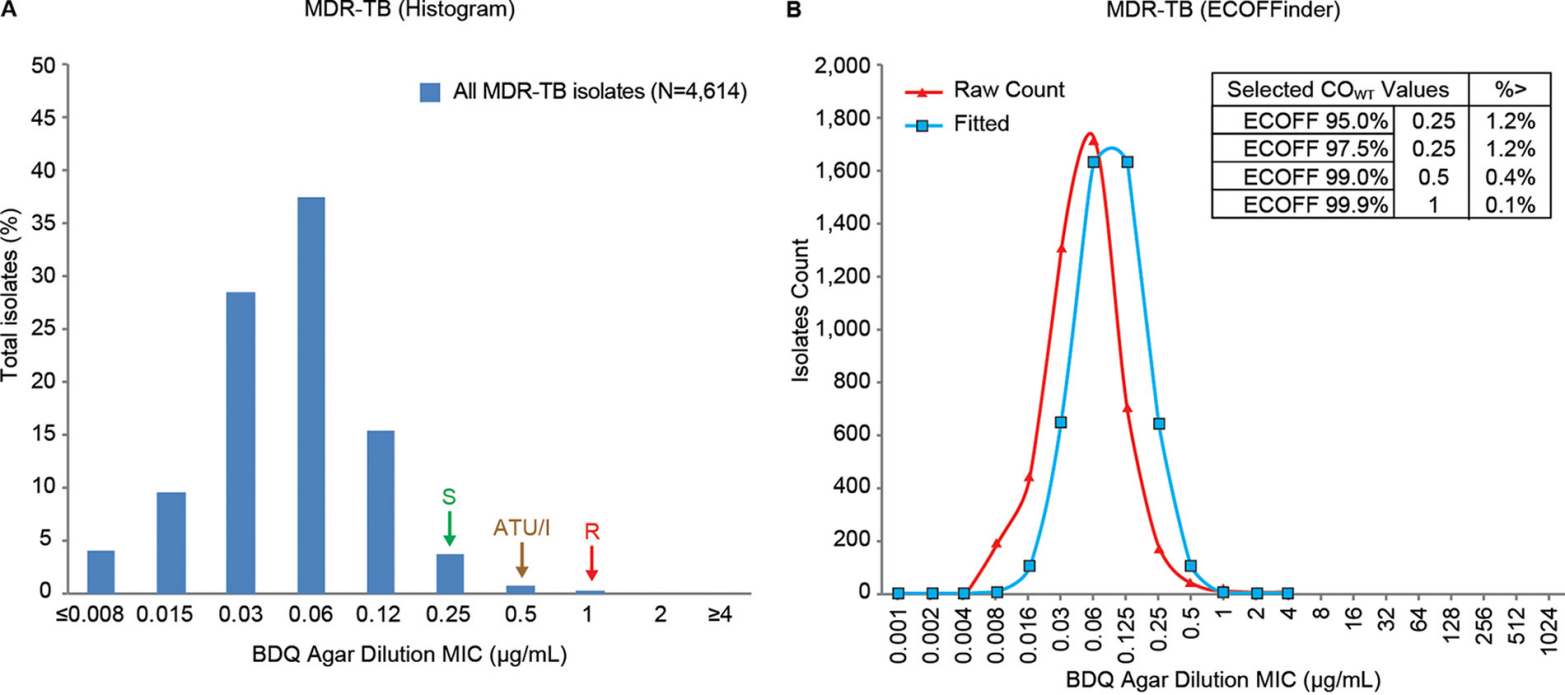
Bedaquiline agar dilution MIC distribution for MDR-TB clinical isolates. Susceptible (S), area of technical uncertainty (ATU)/intermediate (I), and resistant (R) breakpoints are indicated by arrows.

ECOFFinder-derived ECVs at the 97.5% rate were identical for 7H10 and 7H11 agar (0.25 μg/ml). Hence, these were combined and analyzed as agar. The BDQ AD MIC distribution for all MDR-TB resistance isolates was uniform, with a trailing tail and a peak at 0.06 μg/ml (Fig. 3A), which was the same for the H37Rv distribution (Fig. 1B). An ECV of 0.25 μg/ml was determined from the histogram (Fig. 3A, indicated by S), which was consistent with the 97.5% ECV (0.25 μg/ml) determined from ECOFFinder (Fig. 3B), confirming previous findings (27). Based on this analysis, 1.2% of isolates had MICs above the ECV. MIC distribution by agar type and by country is shown in Table S2 in the supplemental material.

### Definitive interpretive criteria for BDQ phenotypic DST.

Based on data from the current study, the QC parameter for BDQ AD MIC is unchanged compared to the Tier-2 study (25). However, from the current data set the QC range is 0.015 to 0.12 μg/ml for the BDQ BMD (Table 1). Based on the totality of the data from this study and the EQA study (27), ECVs for BDQ are 0.12 μg/ml (BMD assay) and 0.25 μg/ml (AD assay) (Table 2). Confirmatory pDST by MGIT and WGS resulted in an ATU of 0.25 μg/ml (BMD assay) and 0.5 μg/ml (AD assay).

**TABLE 1:** Tier-3 bedaquiline quality control parameters for H37Rv with the 7H9 broth microdilution and agar dilution methods

Bacterium	BDQ MIC (μg/ml) by test medium	
	7H9 broth	7H10/7H11 agar
M. tuberculosis H37Rv	0.015–0.12	0.015–0.12

**TABLE 2:** Definitive bedaquiline interpretive criteria by the 7H9 broth microdilution and agar dilution methods

Test medium	BDQ MIC (μg/ml)		
	S	ATU/I	R
7H9 broth	≤0.12	0.25	≥0.5
7H10/7H11 agar	≤0.25	0.5	≥1

A correlation between BDQ MICs by AD and BMD methods showed an essential agreement of 85%, below the 90% threshold for the methods to be considered essentially identical (37).

**FIG 4:**
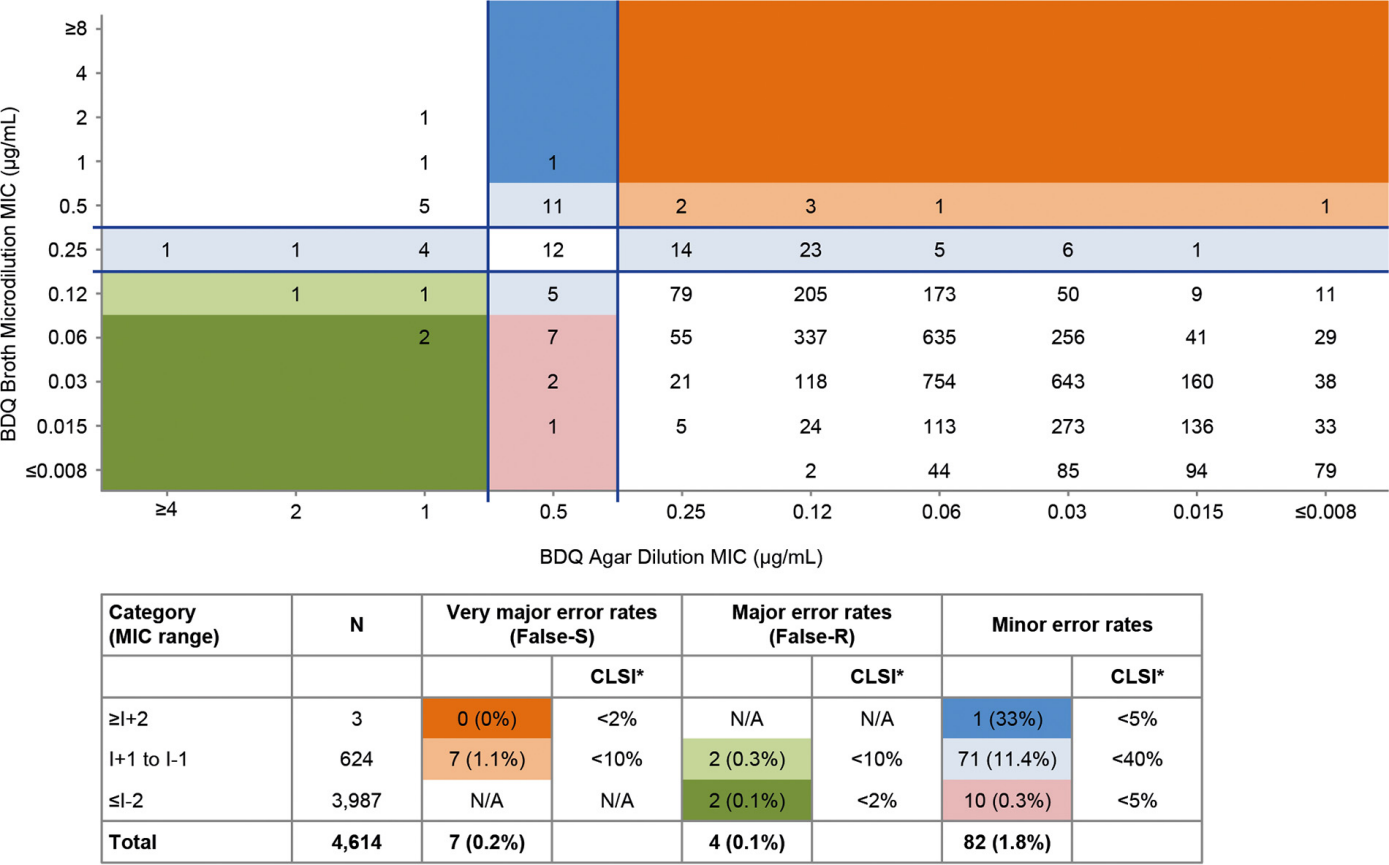
Categorical analysis of bedaquiline breakpoints using the error-rate-bound method. CLSI* indicates guidelines acceptable discrepancy rates (37), NA, not applicable; R, resistant; S, susceptible; I, intermediate. BDQ broth MIC S and R categories are indicated below the lower horizontal line and above the upper horizontal line, respectively. The BDQ agar MIC S and R categories are indicated to the right of the right-hand vertical line and to the left of the left-hand vertical line, respectively. The very major error rates (false susceptible by the agar method) are calculated in the upper right quadrant, where category ≥*I* + 2 is highlighted in dark orange and category *I* + 1 to *I* − 1 is highlighted in light orange. The major error rates (false resistant by the agar method) are calculated in the lower left quadrant, where category *I *+ 1 to *I* − 1 is highlighted in light green and category ≤*I* − 2 is highlighted in dark green. The minor error rates are calculated between the parallel lines, where category ≥*I* + 2 is highlighted in dark blue, category *I* + 1 to *I* − 1 is highlighted in light blue, and category ≤*I* − 2 is highlighted in pink.

An analysis of categorical agreement of BDQ AD and BMD MIC breakpoints, for all isolates having AD and BMD data available (*N* = 4,614) using the error-rate-bound method (Fig. 4), showed that false resistant rates (major error), false susceptible rates (very major error), and minor errors driven by ATU/I are below the CLSI acceptability rates (37).

### BDQ MIC_95_ and ECV-based resistance profile.

The BDQ BMD MIC range for all MDR-TB resistance subtypes combined (*N* = 5,036) was ≤0.008 to 2 μg/ml, although a single isolate with a MIC of 2 μg/ml was identified. Applying the 0.12 μg/ml breakpoint to the final dataset, BDQ-S, BDQ ATU/I, and BDQ-R of all MDR-TB isolates combined were 97.9%, 1.5%, and 0.6%, respectively (Table 3).

**TABLE 3:** Bedaquiline MICs against M. tuberculosis isolates based on subtypes of resistance to other antituberculous drugs^*a*^

Resistance subtype and DST medium	*N*	BDQ MIC (μg/ml)				Susceptibility (%)		
		MIC range	MIC_90_	MIC_95_	ECV	S	ATU/I	R
7H9 broth								
DS-TB	137	≤0.008–0.5	0.12	0.12	0.12	97.8	1.5	0.7
MDR-TB (all)	5,036	≤0.008–2	0.12	0.12	0.12	97.9	1.5	0.6
MDR_H&R_-TB	2,969	≤0.008–0.5	0.12	0.12	0.12	98.1	1.6	0.3
pre-XDR_FQ_-TB	1,155	≤0.008–1	0.12	0.12	0.12	97.9	1.2	0.8
pre-XDR_SI_-TB	277	≤0.008–0.5	0.12	0.12	0.12	98.2	1.1	0.7
XDR-TB	635	≤0.008–2	0.12	0.12	0.12	97.0	1.7	0.9
Agar								
DS-TB	100	≤0.008–0.12	0.06	0.12	0.25	100		0
MDR-TB (all)	4,614	≤0.008–≥4	0.12	0.12	0.25	98.8	0.8	0.4
MDR_H&R_-TB	2,640	≤0.008–1	0.12	0.12	0.25	99.0		1
pre-XDR_FQ_-TB	1,103	≤0.008–1	0.12	0.25	0.25	99.0		1.0
Pre-XDR_SI_-TB	256	≤0.008–1	0.12	0.12	0.25	98.8		1.2
XDR-TB	615	≤0.008–≥4	0.12	0.25	0.25	97.6		2.4

a DST, drug susceptibility testing; DS-TB, drug-susceptible tuberculosis; ECV, epidemiological cutoff value; MDR_H&R_-TB, MDR-TB limited to isoniazid and rifampin resistance; MIC_90_, MIC required to inhibit the growth of 90% of M. tuberculosis isolates; MIC_95_, MIC required to inhibit the growth of 95% of M. tuberculosis isolates; pre-XDR_FQ_-TB, pre-extensively drug (fluoroquinolone)-resistant tuberculosis (MDR-TB with resistance to any fluoroquinolone); pre-XDR_SI_-TB, pre-extensively drug (second-line injectable)-resistant tuberculosis (MDR-TB with resistance to any second-line injectable); XDR-TB, extensively drug-resistant tuberculosis (MDR-TB with resistance to any fluoroquinolone and any second-line injectable).

The BDQ AD MIC range for all MDR-TB resistance subtypes combined (*N* = 4,614) was ≤0.008 to ≥4 μg/ml with a MIC required to inhibit the growth of 90% (MIC_90_) and 95% (MIC_95_) of M. tuberculosis isolates of 0.12 μg/ml. The MIC_95_ was 0.12 μg/ml for MDR_H&R_-TB and pre-XDR_SI_-TB and 0.25 μg/ml for pre-XDR_FQ_-TB and XDR-TB isolates (Table 3). Figure S2 shows there were no trends for decreased BDQ susceptibility against MDR-TB isolates over a 5-year time period from 2015 to 2019.

### Assessment of resistance to fluoroquinolones, second-line injectables and other anti-TB drugs (BMD MIC method). (i) Tier 3-QC values for the drugs tested.

Overall, the number of repeats of the drugs tested against M. tuberculosis H37Rv was higher than the Tier-2 QC ranges (26) (539 versus 211). For most drugs, the frequencies of distribution were still >95%. QC ranges were adjusted for 4/11 drugs (Table 4, in boldface) compared with the Tier-2 QC ranges (26).

**TABLE 4:** QC parameters for other TB drugs for H37Rv by the 7H9 broth microdilution MIC method

Drug name	Drug abbreviation	Tier-2 QC range^*a*^ (μg/ml)	% Repeats within Tier-2 QC range	Tier-3 QC range^*b*^ (μg/ml)	% Repeats within Tier-3 QC range
Rifampicin	RMP	0.03–0.25	93.5	**0.03–0.5**	99.1
Isoniazid	INH	0.03–0.12	95.4	0.03–0.12	95.4
Ethambutol	EMB	0.25–2	94.4	**0.5–4**	96.7
Ofloxacin	OFX	0.25–2	97.2	0.25–2	97.2
Levofloxacin	LVX	0.12–1	99.6	**0.25–1**	98.7
Moxifloxacin	MXF	0.06–0.5	97.6	0.06–0.5	97.6
Kanamycin	KAN	0.25–2	49.7	**0.5–4**	98.3
Amikacin	AMI	0.25–2	98.1	0.25–2	98.1
Capreomycin	CAP	0.5–4	97.0	0.5–4	97.0
Linezolid	LZD	0.25–2	99.8	0.25–2	99.8
Clofazimine	CFZ	0.03–0.25	83.7	0.03–0.25	83.7

a Data are from Kaniga et al. (26).

b Tier-3 QC ranges in boldface indicate those revised compared with the Tier-2 QC ranges (26).

### (ii) Fluoroquinolone resistance profile.

Ofloxacin (OFX), levofloxacin (LVX), and moxifloxacin (MXF) MIC distributions for all MDR-TB resistance subtypes combined were bimodal (Fig. S3A to C). For the fluoroquinolone resistance profile, MDR_H&R_-TB represents the wild-type population and XDR-TB the resistant population; thus, the use of ECOFFinder was deemed unnecessary. Resultant ECVs were 2 μg/ml, 1 μg/ml, and 0.5 μg/ml for OFX, LVX, and MXF, respectively, which are in line with the *in vitro* and *in vivo* potency of the fluoroquinolones (OFX < LVX < MXF) (Table S3).

### (iii) Second-line injectable resistance profile.

MIC distributions for KAN, amikacin (AMI), and CAP (Fig. S3D to F) all displayed a bimodal distribution profile. As for fluoroquinolones, the wild-type population (MDR_H&R_-TB) was clearly separated from the resistant population (XDR-TB), so ECOFFinder was not used.

For KAN, AMI, and CAP, ECVs of 4 μg/ml, 2 μg/ml, and 4 μg/ml, respectively, delineated the wild-type population from the non-wild-type population, with nearly 100% coverage of MDR_H&R_-TB and pre-XDR_FQ_-TB (Table S4).

### (iv) Linezolid and clofazimine resistance profile.

The MIC distributions for LZD and CFZ (Fig. S3G and H) were not bimodal. The trailing MICs at the upper end of the distributions made it difficult to pinpoint an ECV for both drugs. The probable ECVs of 2 μg/ml and 0.5 μg/ml were set for LZD and CFZ, respectively (Table S5). LZD-sensitive and clofazimine-sensitive (CFZ-S) rates of MDR-TB clinical isolates determined from the histograms were 98.5% and 98%, respectively (Table S5).

Because of the trailing MIC issues and the fact that no resistance subtypes were predefined for LZD and CFZ, the ECOFFinder tool was used to derive ECVs of 2 μg/ml for LZD and 0.25 μg/ml for CFZ at 97.5% cutoff, which were identical and lower, respectively, than values determined by the histogram (Table S5). However, the ECOFFinder-derived ECV for CFZ would split the normal MIC, so an ECV of 0.5 μg/ml was selected based upon the histogram, corresponding to 99% coverage by the ECOFFinder.

### (v) Proposed breakpoints for other TB drugs against MDR-TB by the 7H9 broth microdilution MIC method.

The ECVs determined for fluoroquinolones, second-line injectables, LZD, and CFZ are summarized in Table 5 by the BMD MIC method.

**TABLE 5:** Proposed interpretive criteria for other TB drugs based on ECVs for MDR-TB isolates by the 7H9 broth microdilution method^*a*^

Drug name	Drug abbreviation	MIC (μg/ml)	
		S	R
Rifampicin	RMP	NA	NA
Isoniazid	INH	NA	NA
Ethambutol	EMB	NA	NA
Ofloxacin	OFX	2	4
Levofloxacin	LVX	1	2
Moxifloxacin	MXF	0.5	1
Kanamycin	KAN	4	8
Amikacin	AMI	2	4
Capreomycin	CAP	4	8
Linezolid	LZD	2	4
Clofazimine	CFZ	0.5	1

a ECV, epidemiological cutoff value; NA, not applicable because only MDR-TB isolates were tested.

### (vi) Contribution of countries’ BDQ-, CFZ-, and LZD-resistant isolates in the XDR-TB subsets.

As the overall resistance rate of XDR-TB isolates to BDQ (3.0% [19/635]) was 2.5 times lower than to CFZ (7.4% [47/635]) and LZD (7.4% [47/635]), we investigated whether there may be country-specific differences in the prevalence of resistance to BDQ, CFZ, and LZD. Overall, 11/19 (57.9%) and 5/19 (26.3%) isolates resistant to BDQ originated from South Africa and Lithuania, respectively, with 13/47 (27.7%) and 32/47 (68.1%) isolates resistant to CFZ, respectively, originating from these countries. For LZD, 22/47 (46.8%) and 12/47 (25.5%) isolates resistant to LZD originated from Thailand and South Africa, respectively.

### Evaluation of potential cross-resistance between BDQ and CFZ, coresistance to BDQ and LZD, and coresistance to CFZ and LZD. (i) Cross-resistance between BDQ and CFZ.

**FIG 5:**
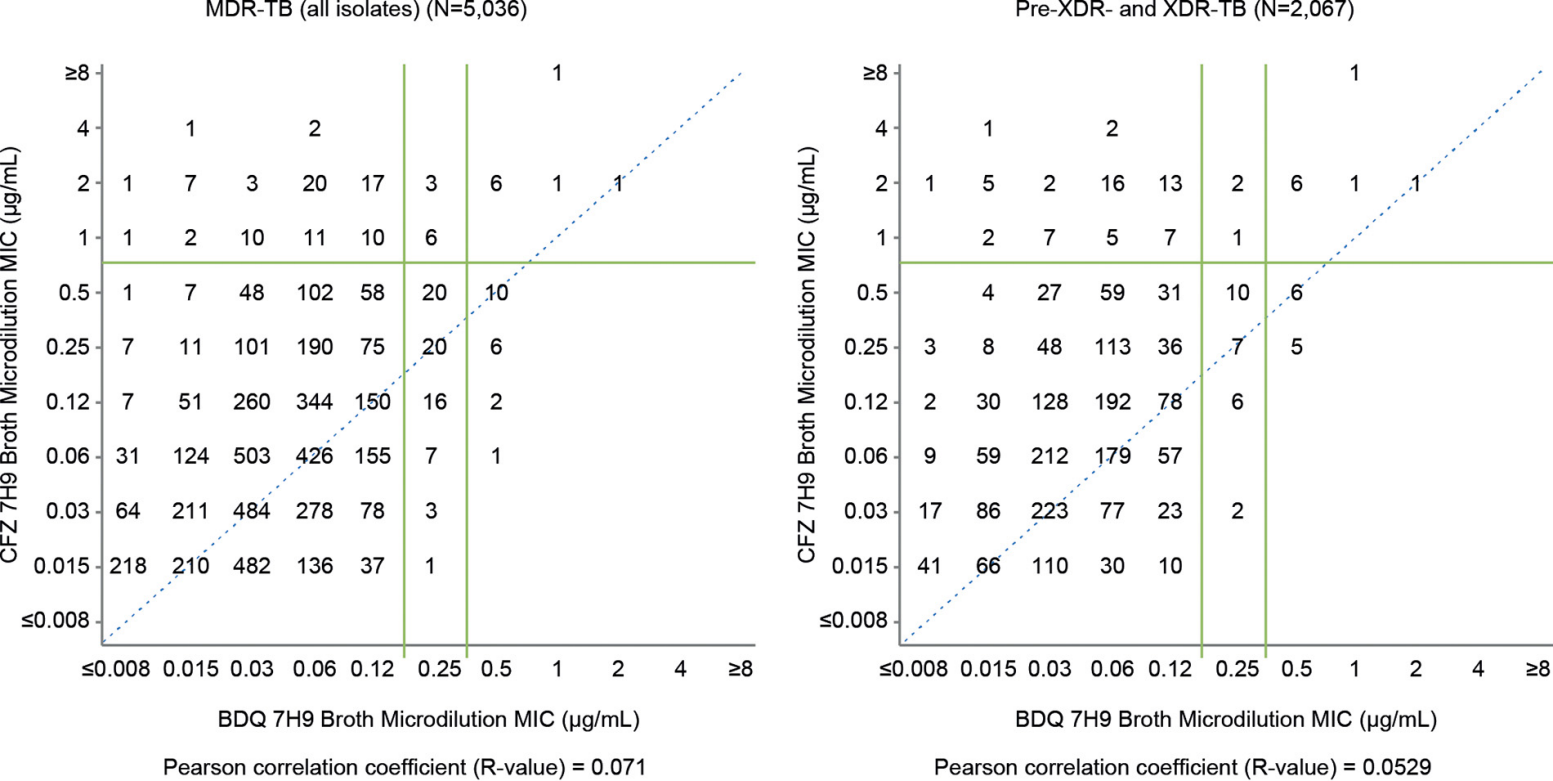
Cross-resistance between bedaquiline and clofazimine. Pre-XDR-TB, pre-extensively drug-resistant tuberculosis (MDR-TB with resistance to any fluoroquinolone or second-line injectable); XDR-TB, extensively drug-resistant tuberculosis (MDR-TB with resistance to any fluoroquinolone and any second-line injectable).

Applying the putative breakpoints defined as ECVs in this study, the potential one-way cross-resistance between BDQ and CFZ was 1.7% (85/5036) BDQ-S and clofazimine resistant (CFZ-R) in the MDR-TB population and 1.8% (89/5036) were CFZ-S and BDQ-R. In the total MDR-TB population, 16.8% (18/107) of the BDQ-R subpopulation was CFZ-R, and 17.5% (18/103) of the CFZ-R subpopulation was BDQ-R. In the pre-XDR-TB/XDR-TB population, proportions were 24% (12/50) and 16.4% (12/73), respectively. Two-way cross-resistance between BDQ and CFZ in the total MDR-TB population was 0.4% (18/5,036) and in the pre-XDR-TB/XDR-TB subset was 1.0% (12/2,067). Scattergrams of BDQ MICs versus CFZ MICs indicated a poor correlation of any cross-resistance in both all MDR-TB isolates (*N* = 5,036; Pearson correlation coefficient *R* value of 0.071) and pre-XDR- and XDR-TB isolates (*N *= 2,067; *R* value of 0.0529) (Fig. 5).

### (ii) Coresistance to BDQ and LZD and to CFZ and LZD.

As BDQ and LZD have been elevated to WHO group A and CFZ to group B, we determined the level of coresistance to BDQ and LZD and to CFZ and LZD. As expected, coresistance to BDQ and LZD (0.1% [5/5,036] in the total MDR-TB population and 0.2% [5/2,067] in the pre-XDR-TB/XDR-TB subset) was very low. Coresistance to CFZ and LZD in the respective populations (0.3% [14/5,036] and 0.4% [9/2,067]) was also very low. Simultaneous resistance to BDQ, CFZ, and LZD was seen in 3/5,036 isolates (0.06%), all from South Africa (2 XDR-TB from Eastern Cape, 1 XDR-TB from Gauteng).

## DISCUSSION

The DREAM study was a prospective *in vitro* study conducted in 11 countries over a 5-year period after BDQ approval to determine the level of susceptibility of MDR-TB isolates to BDQ using the MIC pDST methodology. As expected, based on both BMD and AD methods, high susceptibility rates (≥97%) of MDR-TB, pre-XDR-TB, and XDR-TB isolates to BDQ were seen, since the isolates in this study were recovered from BDQ treatment-naive patients. There were no changes in BDQ susceptibility against MDR-TB isolates based on BDQ BMD MIC distribution over 5 years.

The study determined that the BDQ agar dilution MIC QC range for the H37Rv strain is unchanged (0.015 to 0.12 μg/ml) compared with the Tier-2 study (25) and identified a new BDQ BMD MIC QC range of 0.015 to 0.12 μg/ml. Based on this study and the EQA study (27), BDQ ECVs are 0.12 μg/ml for the BMD MIC and 0.25 μg/ml for the AD MIC. These breakpoints are consistent with putative values determined in the phase 2 BDQ studies (7, 8), although these studies included a limited number of patients with high-MIC isolates. It is important to note that ECVs are not the same as clinical breakpoints, which are a combination of the microbiological cutoff, pharmacokinetic parameters, pharmacokinetic/pharmacodynamic cutoff (PC), and CC.

Given the essential agreement of 85%, this study also confirms previous findings that AD and BMD pDST are not identical for BDQ (25), which justifies the different interpretative criteria for those methods. Categorical analysis of BDQ breakpoints using the error rate-bound method demonstrated that when performing BDQ pDST by the AD method, some isolates may be falsely reported as sensitive to BDQ when in fact they might be phenotypically resistant. Hence, when isolates are reported as BDQ sensitive by the AD MIC method and the MIC is close to the ECV of 0.25 μg/ml, it is recommended that one repeats BDQ pDST by the MGIT method, which is the most reliable pDST method for BDQ.

The prevalence of phenotypic cross-resistance between BDQ and CFZ in the current study was very low, only 0.4% in 5,036 isolates from a BDQ- and CFZ-naive overall MDR-TB population and only 1% in the pre-XDR-TB/XDR-TB subset. Although the risk for emergence of resistance should not be dismissed, currently available clinical data support this observation. A recent study found no statistical correlation between CFZ and BDQ MICs (38). Patients treated with BDQ-containing regimens achieved a comparable sputum culture conversion rate regardless of baseline CFZ susceptibility. In addition, baseline CFZ resistance had no influence on time to culture conversion in their cohort. Acquired CFZ resistance emerged in 8/94 cases, 8.5%, during treatment for MDR-TB, and 5/8 CFZ-R cases achieved culture conversion by completing 24 weeks of therapy containing BDQ (38).

While small *in vitro* and clinical studies have shown BDQ-CFZ cross-resistance and raised concerns that the effectiveness of BDQ against MDR-TB could be impaired when *Rv0678* RAVs are found (14, 20–23), adequately powered studies, such as our current study, with large numbers of patients have increasingly shown evidence of BDQ overcoming cross-resistance to CFZ, achieving more satisfactory clinical outcomes in treating MDR-TB patients. At this time, there are insufficient data from adequately sized clinical studies or treatment cohorts that indicate that the presence of *Rv0678* RAVs consistently leads to poor clinical outcome. Previous data indicate that *Rv0678* RAVs are not associated with prior BDQ or CFZ use, may not lead to elevated BDQ MICs above the breakpoint (≥0.25 μg/ml), and may not be correlated with increased microbiologic failures (18, 21, 39). In another recent study (40), 6/277 (2.2%) patients had BDQ phenotypically resistant isolates prior to receiving BDQ; sputum culture conversion was achieved in 5/6 patients, and 3/6 harbored *Rv0678* RAVs. Existing mutations in the *Rv0678* gene did not predict poor outcome in this limited data set. This issue is further confounded when *Rv0678* is also found in BDQ phenotypically susceptible isolates, as shown in Liu et al. (40) and the current study. In our study, for isolates with BDQ BMD MIC of 0.25 μg/ml, 37.8% were BDQ-R by MGIT, among which 58.8% had *Rv0678* RAVs and 41.2% were wild type for *Rv0678*. The remaining 62.2% were BDQ-S by MGIT, among which 7.1% had *Rv0678* RAVs and 92.9% were wild type for *Rv0678*. Further investigating the use of *Rv0678* as a genetic marker for resistance prediction should be prioritized. Meanwhile, based upon the totality of available data, we propose that the presence of *Rv0678* mutations cannot be used to make clinical decisions to initiate or halt BDQ treatment. When *Rv0678* mutations are found during treatment, we suggest MGIT pDST should be performed, and the patients’ clinical presentation should be the guiding principle for clinicians.

Our study suggests that prior BDQ use may not explain the origin of *Rv0678* RAVs as all patients were BDQ treatment naive, and prior use of CFZ may not be the main reason for the presence of these mutations in the MDR-TB population. Their origin remains unknown (18), although spontaneous mutations are sometimes observed in the absence of drugs. Prior CFZ use should also not be used to exclude BDQ treatment. Development of resistance is expected for any antimicrobial drug, especially when the drug is given in inappropriate or weak regimens. Importantly, the criterion that should be relied upon currently to inform decisions for an appropriate regimen is the phenotypic resistance to BDQ or CFZ.

The prevalence of coresistance to BDQ and LZD and to CFZ and LZD was also very low in both the overall MDR-TB and pre-XDR-TB/XDR-TB populations. Previous studies have not found specific mutations associated with resistance to BDQ and LZD (19, 41, 42) or resistance to CFZ and LZD (22, 42).

Our study also determined that for 4 out of the 11 other anti-TB drugs (rifampicin, ethambutol, LVX, and KAN) evaluated in this study, adjustments were required for the Tier-2 study MIC QC ranges (26). Overall, the ECVs for the fluoroquinolones and second-line injectables tested in our study correlated well with results reported in a single-country (South Africa) study using the Sensititre BMD assay (43), except for KAN (4 versus 8 μg/ml, respectively) and CFZ (0.5 versus 0.25 μg/ml, respectively). The ECV for KAN in the South African study may be higher due to more XDR-TB isolates and identified *eis* mutations than in our study samples. The difference for CFZ ECV between the studies may be explained by the trailing MIC in our study and, as such, 0.5 μg/ml could be considered a conservative option. Although the resistance rates of XDR-TB to KAN, AMI, and CAP were slightly lower than those for the fluoroquinolones in our study, use of second-line injectables was deprioritized in the most recent WHO guidelines (44). Resistance rates to LZD and CFZ were low and similar to those of BDQ.

The overall number of XDR-TB isolates resistant to BDQ, CFZ, or LZD was low. Nonetheless, there seemed to be a trend for resistance to BDQ and CFZ in South Africa and Lithuania and for resistance to LZD in South Africa and Thailand. In South Africa, these observations may be explained by the history of BDQ, CFZ, and LZD use through the BDQ Compassionate Use Access Program and the National TB Treatment Guidelines. At the time of the study CFZ was not used in Lithuania, so the origin of the high CFZ resistance rate in the XDR-TB isolates is unknown. In Thailand, LZD has been used in the country for approximately 15 years in methicillin-resistant Staphylococcus aureus treatment, although this use is considered unlikely to have an impact on M. tuberculosis, as the treatment is usually of short duration. The trend for higher resistance in XDR-TB remains unexplained. The possibility of inaccurate ECVs, leading to some XDR isolates being falsely reported as phenotypically resistant to BDQ, CFZ, or LZD, cannot be ruled out.

One limitation of the study was that it only included BDQ treatment-naive patients. This would have allowed for comparison of the MIC QC ranges and ECVs for BDQ in DREAM with those obtained previously in the Tier-2 study (25, 26; see also the supplemental material) and EQA study (27), respectively. Inclusion of isolates from patients who had failed on a BDQ treatment regimen and were clinically resistant to BDQ would also have enabled the comparison of the MIC values of these resistant strains to those from BDQ treatment-naive patients. Another limitation is that it was not possible to demonstrate the origin of the high CFZ-R rate in XDR-TB isolates in Lithuania. While clustered analyses and repeat DST at another center/country may have answered this question, it was beyond the scope of the study, as no genotyping was initially planned as part of the protocol.

In conclusion, resistance rates to BDQ in the period 2015 to 2019 appeared to be low in the BDQ treatment-naive population, as expected given the early phase of drug introduction to the market. Moreover, no treatment-limiting patterns for cross-resistance were identified with key TB drugs to date. Coresistance to BDQ and LZD and to CFZ and LZD were very low in the populations tested. In addition to clinical criteria, pDST testing remains a relevant approach for informing treatment decisions.
