# Supplementary material for: Bedaquiline Drug Resistance Emergence Assessment in Multidrug-Resistant Tuberculosis (MDR-TB): a 5-Year Prospective In Vitro Surveillance Study of Bedaquiline and Other Second-Line Drug Susceptibility Testing in MDR-TB Isolates
Source: J Clin Microbiol. 2022 Jan 19;60(1):e02919-20. doi: 10.1128/JCM.02919-20 (PMC8769720; doi:10.1128/JCM.02919-20)
Supplement: Supplemental file 1 — Text S1, Fig. S1 to S3, and Tables S1 to S5. Download JCM.02919-20-s0001.pdf, PDF file, 0.8 MB [file jcm.02919-20-s0001.pdf]

Supplementary material

Tier-1, Tier-2 and Tier-3 quality control studies

In the diagnostic microbiology laboratory, susceptibility testing requires regular testing of standard quality control (QC) strains against the antimicrobial agents being used in order to ensure test performance (1–4). Methods whereby these ranges are set for their susceptibility testing methods are described by the Clinical and Laboratory Standards Institute (CLSI) (1).

A Tier-1 study or preliminary quality control range study would be conducted for control testing of new quality control strains and performance of susceptibility tests during drug development. A Tier-1 study would usually performed in a single laboratory with a limited number of replicates (1,2).

A Tier-2 quality control reproducibility study is conducted to establish standardized drug susceptibility testing methodologies and reference MIC quality control ranges for a new antimicrobial agent (1–4). A Tier-2 study must involve at least seven independent laboratories, which are required to test the antimicrobial agent in or on three separate lots of medium from two different manufacturers at least 30 times (from 30 separately prepared inocula) (1–4).

Tier-3 quality control monitoring is conducted to reassess or revise the quality control ranges (1–4).

Whole genome sequencing methodology and bioinformatic analysis for variant calling, lineage assignment and phylogenetic analysis

Whole genome sequencing (WGS) was performed on all *Mycobacterium tuberculosis* complex isolated from liquid culture on the MiSeq (Illumina, UK) as previously described (5). Briefly, the Nextera-XT or Nextera DNA Flex library preparation kit (Illumina, UK) was used to perform library preparation followed by sequencing using the 2 x 300bp MiSeq cartridge v.3 (Illumina, UK) with a targeted 100x depth of coverage.

Resequencing analysis was carried out on CLC Genomics Workbench 11.0.2 (Qiagen, Venlo, Netherlands). Single nucleotide polymorphisms (SNPs) were identified by reference mapping of the paired-end reads against the reference genome H37Rv (NC000962.3), with quality-based and coverage filters applied to each SNP using the following parameters Q-score ≥ 20, a frequency of 10% or more provided at least 5 reads supported the snp in both sequence directions (forward and reverse) as evidence supports a ≥5% frequency to most likely be clinically relevant (6), we set 10% as to increase stringency.

FIG. S1: Distribution of isolates per country.

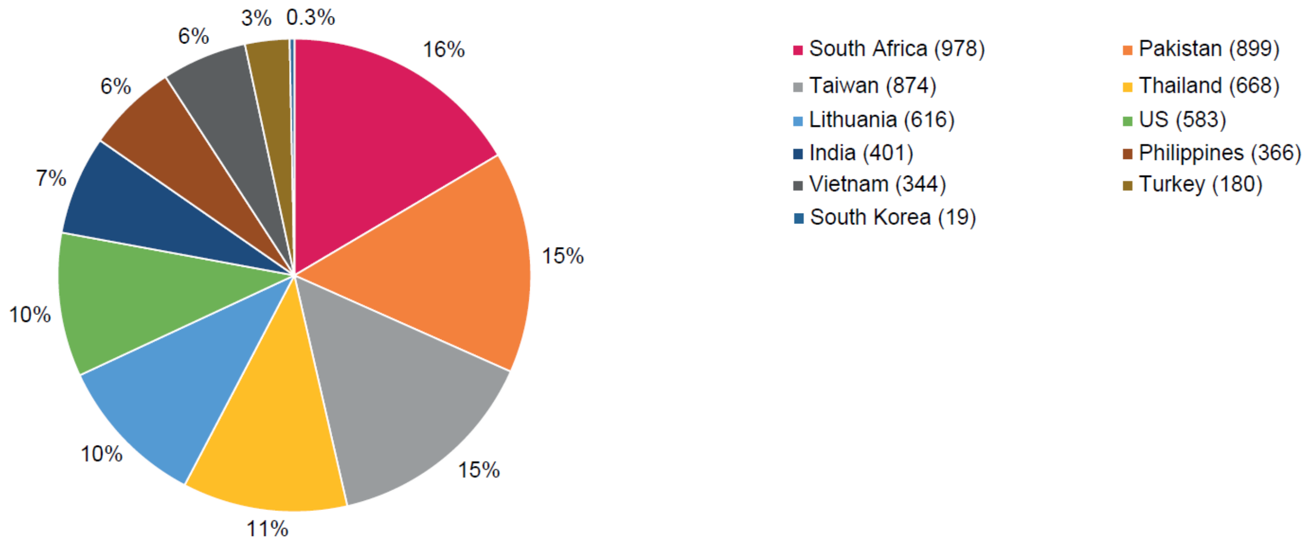

FIG. S2: Evolution of BDQ BMD MIC distribution against MDR-TB over a 5-year period (2015–2019).

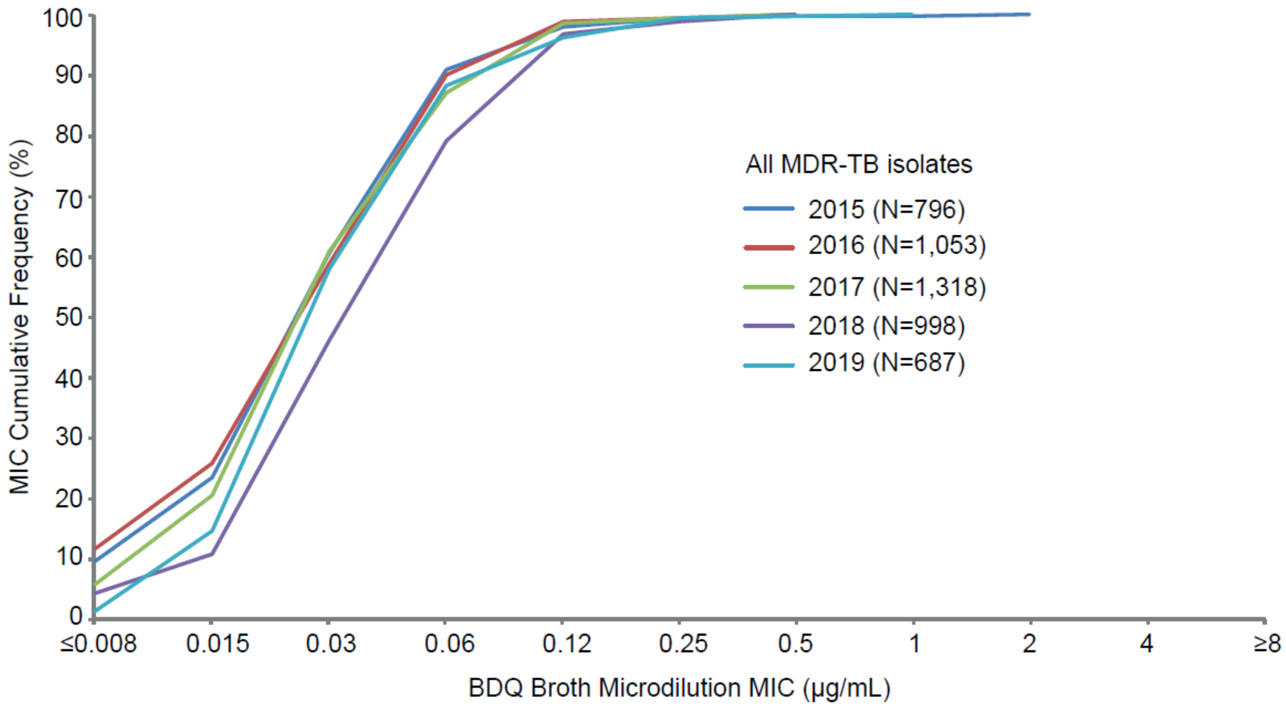

FIG. S3: 7H9 broth microdilution MIC distribution for other anti-TB drugs.

A) Ofloxacin

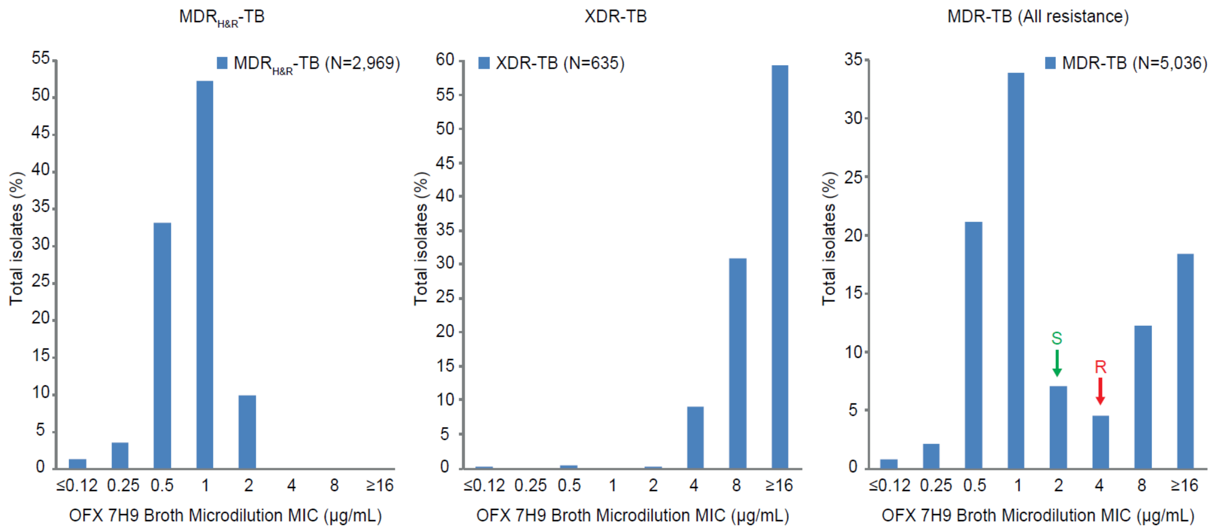

Putative interpretation is indicated by the downward arrow (S and R for susceptible and resistant, respectively).

B) Levofloxacin

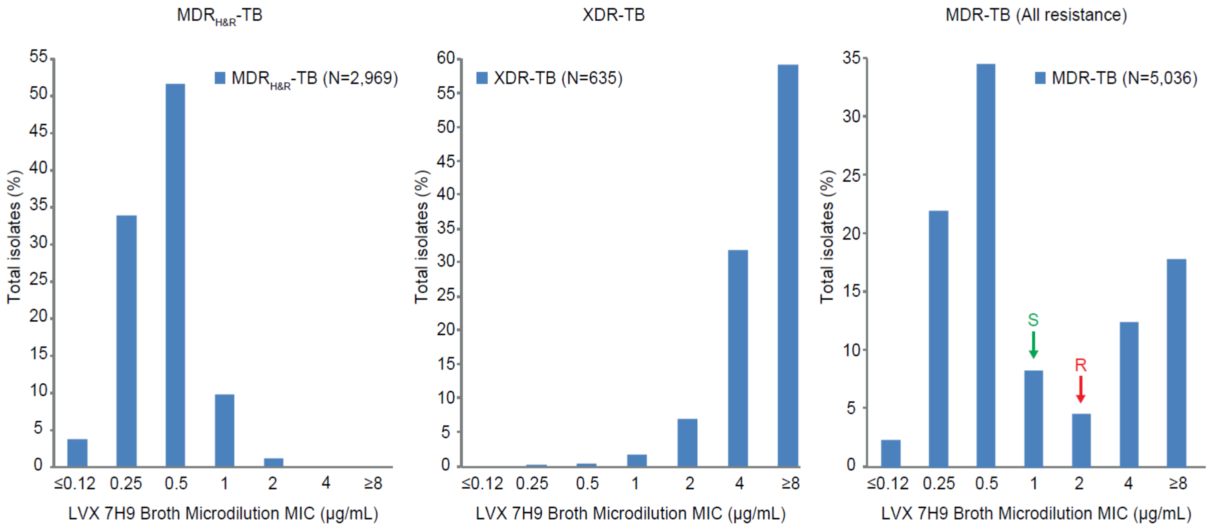

Putative interpretation is indicated by the downward arrow (S and R for susceptible and resistant, respectively).

C) Moxifloxacin

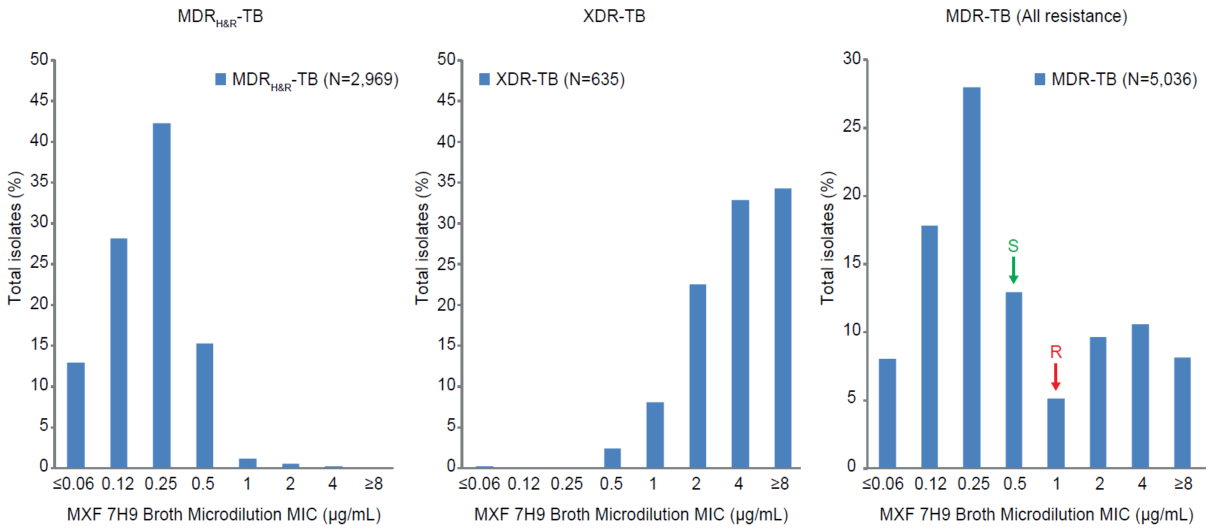

Putative interpretation is indicated by the downward arrow (S and R for susceptible and resistant, respectively).

D) Kanamycin

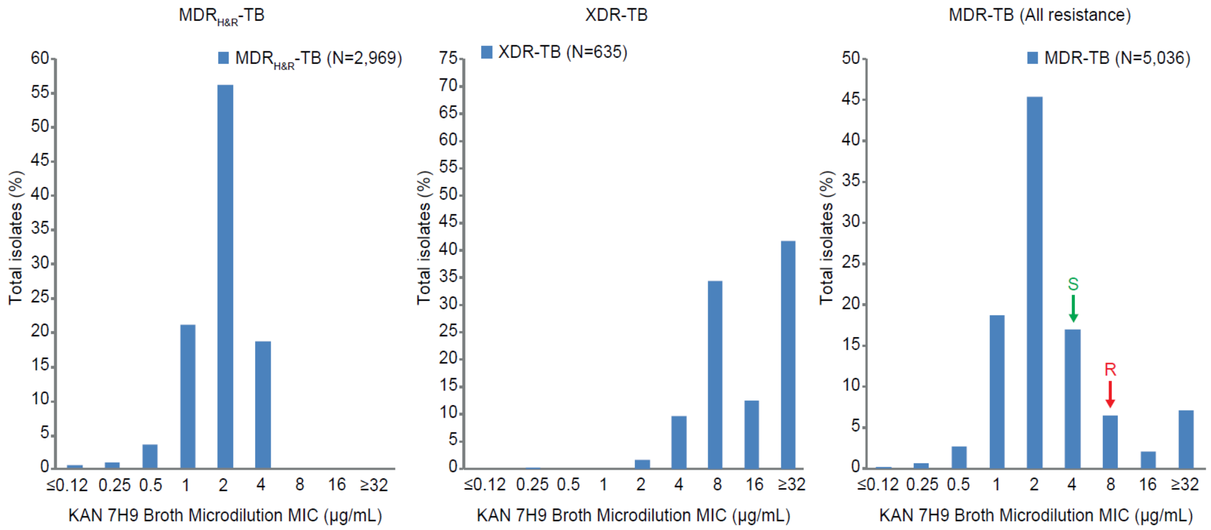

Putative interpretation is indicated by the downward arrow (S and R for susceptible and resistant, respectively).

E) Amikacin

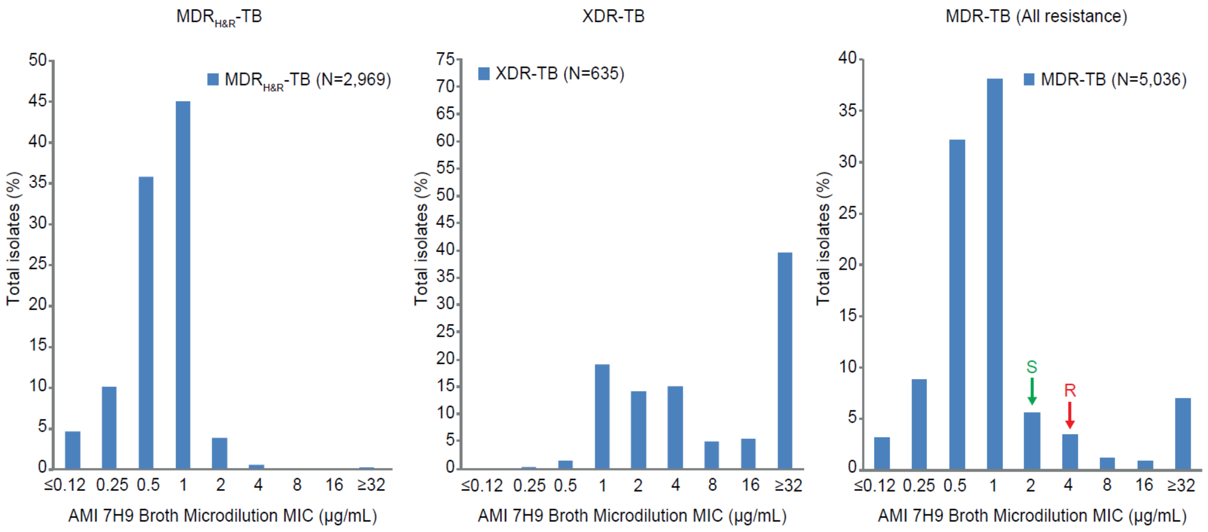

Putative interpretation is indicated by the downward arrow (S and R for susceptible and resistant, respectively).

F) Capreomycin

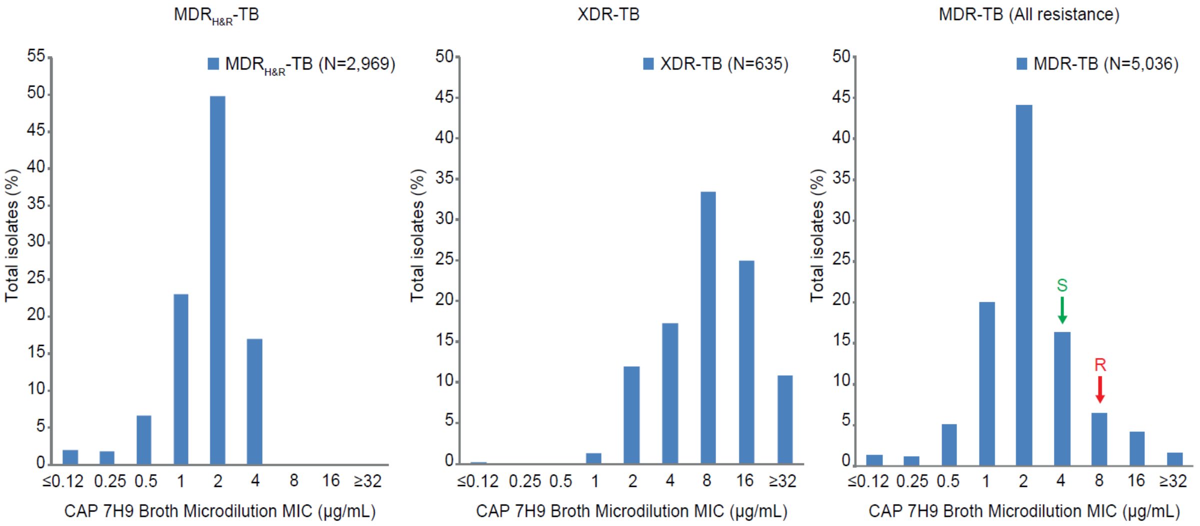

Putative interpretation is indicated by the downward arrow (S and R for susceptible and resistant, respectively).

G) Linezolid

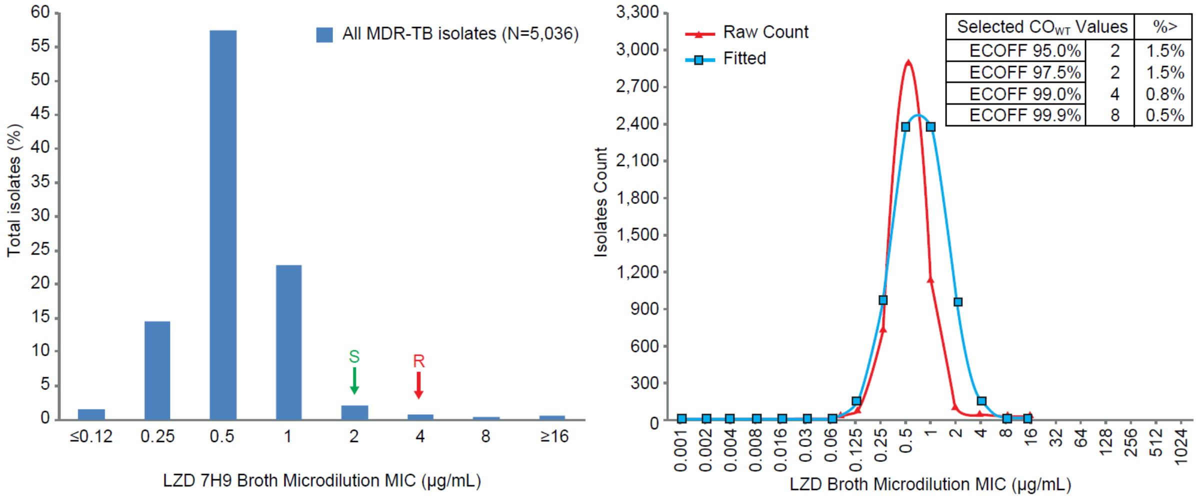

Putative interpretation is indicated by the downward arrow (S and R for susceptible and resistant, respectively).

H) Clofazimine

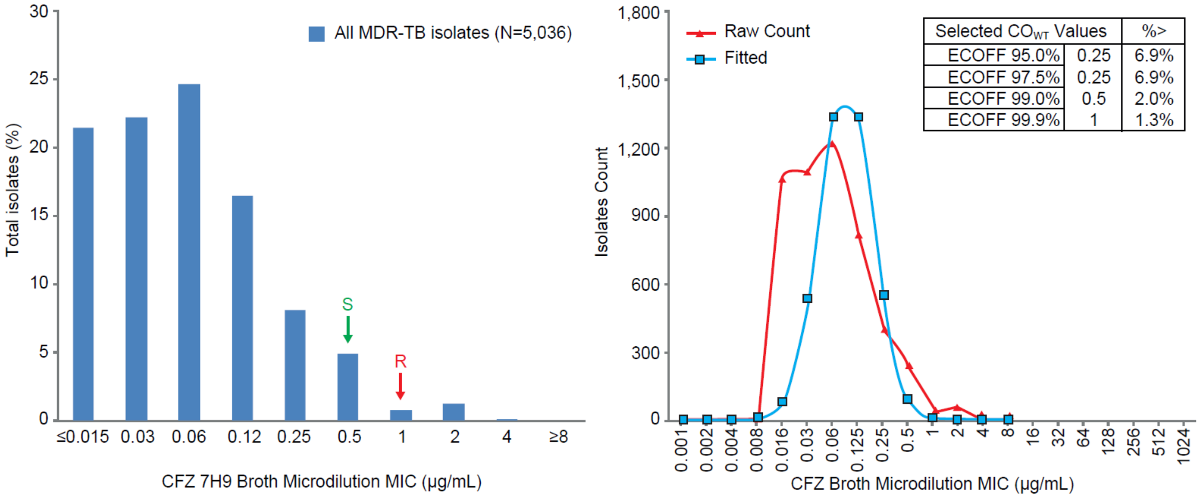

Putative interpretation is indicated by the downward arrow (S and R for susceptible and resistant, respectively).

MDR-TB, multidrug resistant tuberculosis; $MDR_{H\&R}$-TB, MDR-TB limited to isoniazid and rifampin resistance; MIC, minimal inhibitory concentration; XDR-TB, extensively drug resistant tuberculosis (MDR-TB with resistance to any fluoroquinolone and any second-line injectable)

TABLE S1 *Mycobacterium tuberculosis* resistance subtypes in the resazurin microtiter assay

Isolates categories		Programmatic algorithm
**MTB**	All isolates	All isolates tested
**DS-TB**	Sensitive to rifampicin and isoniazid AND to any second-line injectable AND to any fluoroquinolone, to be excluded from all isolates	INH MIC ≤0.25 µg/ml AND RMP MIC ≤0.5 µg/ml<br>In addition<br>LVX MIC ≤2 µg/ml AND OFX MIC ≤2 µg/ml<br>In addition<br>CAP MIC ≤4 µg/ml AND KAN MIC ≤4 µg/ml
**MDR-TB**	All resistance subtypes	INH MIC >0.25 µg/ml AND RMP MIC >0.5 µg/ml
**MDR$_{H\&R}$-TB**	Resistant to ONLY rifampicin AND isoniazid	INH MIC >0.25 µg/ml AND RMP MIC >0.5 µg/ml<br>In addition<br>LVX MIC ≤2 µg/ml AND OFX MIC ≤2 µg/ml<br>In addition<br>CAP MIC ≤4 µg/ml AND KAN MIC ≤4 µg/ml
**Pre-XDR$_{FQ}$-TB**	Resistant to any fluoroquinolone BUT NOT to any second-line injectable	INH MIC >0.25 µg/ml AND RMP MIC >0.5 µg/ml<br>In addition<br>LVX MIC >2 µg/ml OR OFX MIC >2 µg/ml<br>But<br>CAP MIC ≤4 µg/ml AND KAN MIC ≤4 µg/ml
**Pre-XDR$_{SI}$-TB**	Resistant to any second-line injectable BUT NOT to any fluoroquinolone	INH MIC >0.25 µg/ml AND RMP MIC >0.5 µg/ml<br>In addition<br>CAP MIC >4 µg/ml OR KAN MIC >4 µg/ml<br>But<br>LVX MIC ≤2 µg/ml AND OFX MIC ≤2 µg/ml
**XDR-TB**	Resistant to any second-line injectable AND to any fluoroquinolone	INH MIC >0.25 µg/ml AND RMP MIC >0.5 µg/ml<br>In addition<br>LVX MIC >2 µg/ml OR OFX MIC >2 µg/ml<br>In addition<br>CAP MIC >4 µg/ml OR KAN MIC >4 µg/ml

The resazurin microtiter assay critical concentrations for first- and second-line drugs were used to define the MTB resistance subtypes (7–11).

CAP, Capreomycin; DS-TB, drug-susceptible tuberculosis; INH, Isoniazid; KAN, Kanamycin; LVX, Levofloxacin; MDR-TB, multidrug resistant tuberculosis; MIC, minimal inhibitory concentration; MTB, *Mycobacterium tuberculosis;* OFX, Ofloxacin; Pre-XDR$_{FQ}$-TB, pre-extensively drug (fluoroquinolone)-resistant tuberculosis; Pre-XDR$_{SI}$-TB, pre-extensively drug (second-line injectable)-resistant tuberculosis; RMP, Rifampicin; XDR-TB, extensively drug-resistant tuberculosis.

TABLE S2 *Bedaquiline Agar MIC distribution by media type by country against MDR-TB (all resistance subtypes).*

		Bedaquiline (BDQ) 7H11 Agar MIC (μg/mL)										
**Country**	**Statistic**	**≤ 0.008**	**0.015**	**0.03**	**0.06**	**0.12**	**0.25**	**0.5**	**1**	**2**	**≥ 4**	**Total**
				**7H10 MIC Distributions**								
India	N	15	77	110	130	65	2	1				400
	% Total	3.8	19.3	27.5	32.5	16.3	0.5	0.3				
	% Cumulative	3.8	23.0	50.5	83.0	99.3	99.8	100.0				100.0
South Africa	N	56	53	191	262	101	87	19	4	2	1	776
	% Total	7.2	6.8	24.6	33.8	13.0	11.2	2.4	0.5	0.3	0.1	
	% Cumulative	7.2	14.0	38.7	72.4	85.4	96.6	99.1	99.6	99.9	100.0	100.0
Pakistan	N	12	32	226	374	195	46	5	6			896
	% Total	1.3	3.6	25.2	41.7	21.8	5.1	0.6	0.7			
	% Cumulative	1.3	4.9	30.1	71.9	93.6	98.8	99.3	100.0			100.0
				**7H11 MIC Distributions**								
Lithuania	N		11	172	356	34	7	4				584
	% Total		1.9	29.5	61.0	5.8	1.2	0.7				
	% Cumulative		1.9	31.3	92.3	98.1	99.3	100.0				100.0
Philippines	N	27	67	115	75	46						330
	% Total	8.2	20.3	34.8	22.7	13.9						

	% Cumulative	8.2	28.5	63.3	86.1	100.0						100.0
Taiwan	N	4	19	72	243	184	19	7	1			549
	% Total	0.7	3.5	13.1	44.3	33.5	3.5	1.3	0.2			
	% Cumulative	0.7	4.2	17.3	61.6	95.1	98.5	99.8	100.0			100.0
Thailand	N	45	92	264	155	44	5					605
	% Total	7.4	15.2	43.6	25.6	7.3	0.8					
	% Cumulative	7.4	22.6	66.3	91.9	99.2	100.0					100.0
Turkey	N	32	63	48	9	2						154
	% Total	20.8	40.9	31.2	5.8	1.3						
	% Cumulative	20.8	61.7	92.9	98.7	100.0						100.0
Vietnam	N		27	115	121	41	10	3	3			320
	% Total		8.4	35.9	37.8	12.8	3.1	0.9	0.9			
	% Cumulative		8.4	44.4	82.2	95.0	98.1	99.1	100.0			100.0
All Countries	N	191	441	1313	1725	712	176	39	14	2	1	4614
	% Total	4.1	9.6	28.5	37.4	15.4	3.8	0.8	0.3	0.0	0.0	
	% Cumulative	4.1	13.7	42.2	79.5	95.0	98.8	99.6	99.9	100.0	100.0	100.0

TABLE S3 Fluoroquinolone resistance profile

Drug name<br>DST medium	Resistance subtype	N	MIC (μg/ml)				Susceptibility (%)	
			MIC range	MIC_{90}	MIC_{95}	ECV	S	R
OFX<br>7H9 broth	MDR-TB	5036	≤0.12–≥16	≥16	≥16	2	64.9	35.1
	$MDR_{H\&R}$-TB	2969	≤0.12–2	1	2	2	100	0
	Pre-XDR_{FQ}-TB	1155	0.5–≥16	≥16	≥16	2	1.6	98.4
	Pre-XDR_{SI}-TB	277	0.25–2	2	2	2	100	0
	XDR-TB	635	≤0.12–≥16	≥16	≥16	2	0.6	99.4
LVX<br>7H9 broth	MDR-TB	5036	≤0.12–≥8	≥8	≥8	1	65.8	34.2
	$MDR_{H\&R}$-TB	2969	≤0.12–2	1	1	1	99	1
	Pre-XDR_{FQ}-TB	1155	≤0.12–≥8	≥8	≥8	1	7.6	92.4
	Pre-XDR_{SI}-TB	277	≤0.12–2	1	1	1	98.6	1.4
	XDR-TB	635	0.25–≥8	≥8	≥8	1	2.2	97.8
MXF<br>7H9 broth	MDR-TB	5036	≤0.06–≥8	4	≥8	0.5	66.7	33.3
	$MDR_{H\&R}$-TB	2969	≤0.06–4	0.25	0.25	0.5	98.4	1.6
	Pre-XDR_{FQ}-TB	1155	≤0.06–≥8	≥8	≥8	0.5	13.3	86.7
	Pre-XDR_{SI}-TB	277	≤0.06–≥8	0.5	0.5	0.5	96.4	3.6
	XDR-TB	635	≤0.06–≥8	≥8	≥8	0.5	2.5	97.5

Note: Because pre-XDR_{FQ}-TB is defined as 'resistant to any fluoroquinolone and not resistant to any second-line injectable', there are isolates in the pre-XDR_{FQ}-TB and XDR-TB subpopulations that would genuinely be susceptible to a fluoroquinolone at the identified ECV. Conversely, because pre-XDR_{SI}-TB is defined as 'resistant to any second-line injectable and not resistant to any fluoroquinolone', there are isolates in this subpopulation that would genuinely be resistant to a fluoroquinolone at the identified ECV.

DST, drug-susceptibility testing; ECV, epidemiological cut-off value; LVX, Levofloxacin; MDR-TB, multidrug resistant tuberculosis; $MDR_{H\&R}$-TB, MDR-TB limited to isoniazid and rifampin resistance; MIC, minimal inhibitory concentration; MIC_{90}, MIC required to inhibit the growth of 90% of MTB isolates; MIC_{95}, MIC required to inhibit the growth of 95% of MTB isolates; MXF, moxifloxacin; OFX, Ofloxacin; Pre-XDR_{FQ}-TB, pre-extensively drug (fluoroquinolone)-resistant tuberculosis (MDR-TB with resistance to any fluoroquinolone); pre-XDR_{SI}-TB, pre-extensively drug (second-line injectable)-resistant tuberculosis (MDR-TB with resistance to any second-line injectable); R, resistant; S, susceptible; XDR-TB, extensively drug-resistant tuberculosis (MDR-TB with resistance to any fluoroquinolone and any second-line injectable).

TABLE S4: Second-line injectable resistance profile

Drug name<br>DST medium	Resistance subtype	N	MIC (μg/ml)				Susceptibility (%)	
			MIC range	MIC_{90}	MIC_{95}	ECV	S	R
KAN<br>7H9 broth	MDR-TB (All)	5036	≤0.12–≥32	8	≥32	4	84.5	15.5
	$MDR_{H\&R}$-TB	2969	≤0.12–4	4	4	4	100	0
	Pre-XDR_{FQ}-TB	1155	0.25–4	4	4	4	100	0
	Pre-XDR_{SI}-TB	277	1–≥32	≥32	≥32	4	20.6	79.4
	XDR-TB	635	0.25–≥32	≥32	≥32	4	11.5	88.5
AMI<br>7H9 broth	MDR-TB (All)	5036	≤0.12–≥32	4	≥32	2	87.7	12.3
	$MDR_{H\&R}$-TB	2969	≤0.12–≥32	1	1	2	99.2	0.8
	Pre-XDR_{FQ}-TB	1155	≤0.12–≥32	1	2	2	98.1	1.9
	Pre-XDR_{SI}-TB	277	0.25–≥32	≥32	≥32	2	42.2	57.8
	XDR-TB	635	0.25–≥32	≥32	≥32	2	35	65.0
CAP<br>7H9 broth	MDR-TB (All)	5036	≤0.12–≥32	8	16	4	87.9	12.1
	$MDR_{H\&R}$-TB	2969	≤0.12–4	4	4	4	100	0
	Pre-XDR_{FQ}-TB	1155	≤0.12–4	4	4	4	100	0
	Pre-XDR_{SI}-TB	277	0.5–≥32	16	≥32	4	39.4	60.6
	XDR-TB	635	≤0.12–≥32	≥32	≥32	4	30.6	69.4

Note: Because pre-XDR_{SI}-TB is defined as ‘resistant to any second-line injectable and not resistant to fluoroquinolone, there are isolated in the pre-XDR_{SI}-TB and XDR-TB subpopulations that would genuinely be susceptible to a second-line injectable at the identified ECV. Conversely, because pre-XDR_{FQ}-TB is defined as ‘resistant to any fluoroquinolone and not resistant to second-line injectable’ there are isolates in this subpopulation that would genuinely be resistant to a fluoroquinolone at the identified ECV.

AMI, Amikacin; CAP, Capreomycin; DST, drug-susceptibility testing; ECV, epidemiological cut-off value; KAN, Kanamycin; MDR-TB, multidrug resistant tuberculosis; $MDR_{H\&R}$-TB, MDR-TB limited to isoniazid and rifampin resistance; MIC, minimal inhibitory concentration; MIC_{90}, MIC required to inhibit the growth of 90% of MTB isolates; MIC_{95}, MIC required to inhibit the growth of 95% of MTB isolates; Pre-XDR_{FQ}-TB, pre-extensively drug (fluoroquinolone)-resistant tuberculosis (MDR-TB with resistance to any fluoroquinolone); pre-XDR_{SI}-TB, pre-extensively drug (second-line injectable)-resistant tuberculosis (MDR-TB with resistance to any second-line injectable); R, resistant; S, susceptible; XDR-TB, extensively drug-resistant tuberculosis (MDR-TB with resistance to any fluoroquinolone and any second-line injectable).

TABLE S5 Linezolid and clofazimine resistance profile

Drug name<br>DST medium	Resistance subtype	N	MIC (μg/ml)				Susceptibility (%)	
			MIC range	MIC$_{90}$	MIC$_{95}$	ECV	S	R
LZD<br>7H9 Broth	MDR-TB (All)	5036	≤0.12–≥16	1	1	2	98.5	1.5
	MDR$_{H\&R}$-TB	2969	≤≤0.12–8	1	1	2	99.7	0.3
	Pre-XDR$_{FQ}$-TB	1155	≤0.12–≥16	1	1	2	98.3	1.7
	Pre-XDR$_{SI}$-TB	277	≤0.12–2	1	2	2	99.3	0.7
	XDR-TB	635	≤0.12–≥16	2	4	2	92.6	7.4
CFZ<br>7H9 Broth<br>(Histogram)	MDR-TB (All)	5036	≤0.015–≥8	0.25	0.5	0.5	98	2
	MDR$_{H\&R}$-TB	2969	≤0.015–2	0.25	0.25	0.5	99	1
	Pre-XDR$_{FQ}$-TB	1155	≤0.015–4	0.25	0.25	0.5	99.4	0.6
	Pre-XDR$_{SI}$-TB	277	≤0.015–4	0.25	2	0.5	93.1	6.9
	XDR-TB	635	≤0.015–≥8	0.5	1	0.5	92.6	7.4

CFZ, Clofazimine; DST, drug-susceptibility testing; ECV, epidemiological cut-off value; LZD, Linezolid; MDR-TB, multidrug resistant tuberculosis; MDR$_{H\&R}$-TB, MDR-TB limited to isoniazid and rifampin resistance; MIC, minimal inhibitory concentration; MIC$_{90}$, MIC required to inhibit the growth of 90% of MTB isolates; MIC$_{95}$, MIC required to inhibit the growth of 95% of MTB isolates; Pre-XDR$_{FQ}$-TB, pre-extensively drug (fluoroquinolone)-resistant tuberculosis (MDR-TB with resistance to any fluoroquinolone); pre-XDR$_{SI}$-TB, pre-extensively drug (second-line injectable)-resistant tuberculosis (MDR-TB with resistance to any second-line injectable); R, resistant; S, susceptible; XDR-TB, extensively drug-resistant tuberculosis (MDR-TB with resistance to any fluoroquinolone and any second-line injectable).

REFERENCES

1. Clinical and Laboratory Institute Standards. 2008. Development of in vitro susceptibility testing criteria and quality control parameters; approved guideline, 3rd ed. CLSI document M23-A3. Clinical and Laboratory Standards Institute, Wayne, PA.
2. Turnidge J and Bordash G. 2007. Statistical methods for establishing quality control ranges for antibacterial agents in Clinical and Laboratory Standards Institute susceptibility testing. Antimicrob Agents Chemother 51:2483–2488.
3. Kaniga K, Cirillo DM, Hoffner S, Ismail NA, Kaur D, Lounis N, Metchock B, Pfyffer GE, Venter A. 2016. A multilaboratory, multicountry study to determine bedaquiline MIC quality control ranges for phenotypic drug susceptibility testing. J Clin Microbiol 54:2956–2962.
4. Kaniga K, Cirillo DM, Hoffner S, Ismail NA, Kaur D, Lounis N, Metchock B, Pfyffer GE, Venter A. 2016. A multilaboratory, multicountry study to determine MIC quality control ranges for phenotypic drug susceptibility testing of selected first-line antituberculosis drugs, second-line injectables, fluoroquinolones, clofazimine, and linezolid. J Clin Microbiol 54:2963–2968.
5. Omar SV, Joseph L, Said HM, Ismail F, Ismail N, Gwala TL, Ismail NA. 2019. Whole genome sequencing for drug resistance determination in Mycobacterium tuberculosis. Afr J Lab Med 8:801, doi:10.4102/ajlm.v8i1.801.
6. Nimmo C. Brien K, Millard J, Grant AD, Padayatchi N, Pym AS, O'Donnell M, Goldstein R, Breuer J, Balloux F. 2020. Dynamics of within-host Mycobacterium tuberculosis diversity and heteroresistance during treatment. EBioMedicine 55:102747, doi:10.1016/j.ebiom.2020.102747.
7. Martin A, Camacho M, Portaels F, Palomino J. 2003. Resazurin microtiter assay plate testing of *Mycobacterium tuberculosis*: susceptibilities to second-line drugs: rapid, simple, and inexpensive method. Antimicrob Agents Chemother 47:3616–3619.
8. Martin A, Morcillo N, Lemus D, Montoro E, da Silva Telles MA, Simboli N, Pontino M, Porras T, León C, Velasco M, Chacon L, Barrera L, Ritacco V, Portaels F, Palomino JC. 2005. Multicenter study of MTT and resazurin assays for testing susceptibility to first-line anti-tuberculosis drugs. Int J Tuberc Lung Dis 9:901–906.
9. Martin A, Palomino JC, Portaels F. 2005. Rapid detection of ofloxacin resistance in *Mycobacterium tuberculosis* by two low-cost colorimetric methods: resazurin and nitrate reductase assays. J Clin Microbiol 43:1612–1616.
10. Martin A, Paasch F, Docx S, Fissette K, Imperiale B, Ribón W, González LA, Werngren J, Engström A, Skenders G, Juréen P, Hoffner S, Del Portillo P, Morcillo N,

Palomino JC. 2011. Multicentre laboratory validation of the colorimetric redox indicator (CRI) assay for the rapid detection of extensively drug-resistant (XDR) *Mycobacterium tuberculosis*. J Antimicrob Chemother 66:827–833.

11. Palomino JC, Martin A, Camacho M, Guerra H, Swings J, Portaels F. 2002. Resazurin microtiter assay plate: simple and inexpensive method for detection of drug resistance in *Mycobacterium tuberculosis*. Antimicrob Agents Chemother 46:2720–2722.
